# Comparison of Thyroid Gland Sonography Index with Serum Antithyroid Peroxidase, Antithyroglobulin, and Thyroid Function Tests in Patients with Hashimoto Thyroiditis

**DOI:** 10.31661/gmj.v13i.3309

**Published:** 2024-07-28

**Authors:** Fatemeh Eftekharian, Gholamhossein Ranjbar Omrani, Mohammad Hossein Dabbaghmanesh, Reza Sahraei, Mohammad Ali Behnam, Marzieh Bakhshayeshkaram, Mohammad Mahdi Dabbaghmanesh

**Affiliations:** ^1^ Endocrinology Research Center, Shiraz University of Medical Sciences, Shiraz, Iran; ^2^ Internal Medicine Department, Jahrom University of Medical Sciences, Jahrom, Iran; ^3^ Anesthesiology Department, Jahrom University of Medical Sciences, Jahrom, Iran; ^4^ Medical Physics and Engineering Department, Shiraz University of Medical Sciences, Shiraz, Iran

**Keywords:** Hashimoto’s Thyroiditis, Anti-Thyroid Antibody, Thyroid Ultrasound

## Abstract

Background: Ultrasound examination of the thyroid has emerged as a useful diagnostic and prognostic tool, along with measuring serum titers of anti-thyroid peroxidase (TPO), anti-thyroglobulin (Tg), and thyroid hormones, in patients with Hashimoto's thyroiditis. So, we aimed at considering correlations of ultrasonographic, antibodies, and thyroid hormone levels. Materials and Methods: A total of 149 patients (118 females, 31 males; aged 18–60 years; mean age: 38.60 ± 8.03 years) who were diagnosed with Hashimoto's thyroiditis were enrolled in the study. The blood sample was taken to measure serum titers of free T3 (FT3) and T4 (FT4), TSH, anti-TPO, and anti-Tg antibody titers. The thyroid sonography of each patient was classified into one of the five grades by real-time ultrasound (US) based on echogenicity, thyroid size, and thyroid pattern. We evaluated whether there was a correlation between thyroid characteristics observed via ultrasound and serum levels of thyroid hormones, anti-TPO antibodies, and anti-Tg antibodies. Results: Nodular structures were detected in 54 (36.2%) patients (38 micro-nodular and 16 macro-nodular). Echogenicity was recorded as isoechoic in 15(10.07%) and hypoechoic in 119 (79.87%) subjects. Euthyroid subjects had significantly thicker isthmus than overt and subclinical hypothyroid patients (P=0.018). Mean serum TSH, anti-Tg, and anti-TPO antibody titers showed a significant increase in patients with macro-nodules compared to those with micro-nodules and individuals without nodules (P0.05). The thickness of the isthmus had a significant negative correlation with FT4 (P=0.046; r=0.11) and FT3 (P=0.017; r=0.15), respectively. Thyroid autoantibodies had positive significant correlations with different parameters of thyroid volume (P0.05). Conclusions: Thyroid US findings, in addition to serum anti-Tg and anti-TPO antibody titers, might be correlated with the severity and extent of Hashimoto's thyroiditis, but further evaluations are needed.

## Introduction

Hashimoto's thyroiditis, also known as chronic lymphocytic thyroiditis, is an autoimmune disease characterized by anti-thyroid antibodies [1]. It is one of the most common causes of hypothyroidism, which might be subclinical in 90% of patients [2]. While a definitive diagnosis typically involves thyroid gland sampling, this method is seldom employed. Instead, diagnosis relies on clinical observation and the outcomes of biochemical and serological tests [3]. During the clinical examination, the diagnosis is related to the signs and symptoms of hypothyroidism, goiter with a tingling sensation during the physical examination, and a Delphian node [2].

High titers of anti-thyroid peroxidase (anti-TPO) or anti-thyroglobulin (anti-Tg) antibodies in serum confirm the diagnosis; Serum titers of thyroid stimulating hormone (TSH) and free T4 (FT4) hormone measurements and imaging studies, such as the ultrasound (US), are used to diagnose Hashimoto's thyroiditis. High titers of anti-TPO antibodies are found in 95% of patients with Hashimoto's thyroiditis [4]. Moreover, 70% of Hashimoto patients tested positive for anti-Tg antibodies. However, it's noteworthy that anti-Tg antibodies are detected in 5% of the general population. While there's a strong association between the presence of anti-TPO antibodies and Hashimoto's thyroiditis, it remains uncertain whether the elevated levels of anti-TPO or anti-Tg antibodies directly cause the condition or merely indicate ongoing disruption of thyroid cells. [5].

US examination is widely safe, noninvasive, available, easy to use, and less expensive than other imaging methods. Advanced US imaging in recent years has not only been amazing for radiologists but has also fascinated clinicians to use these techniques in their daily clinical practice. Advances in thyroid imaging have considerably improved the diagnosis, treatment, follow-up, and prognosis of high-prevalence thyroid diseases. The sonographic presentation of Hashimoto's thyroiditis includes a wide range of findings. Typically, thyroid US reveals a large, heterogeneous, and hypoechoic thyroid gland. A micronodular pattern strongly supports the detection of Hashimoto's thyroiditis [6]. Thyroid US is an ideal imaging modality in the evaluation of the thyroid gland, and the relationship between decreased echogenicity, or irregular echo pattern in the US, and thyroid dysfunction is well known. The sonographic finding can play a role in the objective identification of active and latent patients with autoimmune diseases. However, the relationship between antibody titers and thyroid sonographic findings is not clear [7]. The classification of grayscale findings in Hashimoto's thyroiditis was created by Sostre and Reyes [8]. Some descriptive classification systems describe follow-up sonographic scans based on sonographic parameters. However, these classification systems have not yet been widely used in clinical practice. The interplay between the action of thyroid hormones and the immune system has been established in physiological and pathological settings. Moreover, evidence suggests the role of hormone replacement treatment in the modulation of the immune response [9].

The findings of this study may contribute to a broader understanding of the agreement of serological and sonographic markers for Hashimoto’s thyroiditis. Most of the available literature on diagnostic studies in the field of Hashimoto’s thyroiditis has been performed in hypothyroid patients receiving thyroid replacement therapy. To do so, further research is needed to compare the correlation of these sonographic findings and the related classification of grades with the severity of the disease. This study aimed to find out whether there is a correlation between thyroid sonographic parameters and autoantibody activity in patients with Hashimoto's thyroiditis in patients who did not receive thyroid hormone replacement therapy. Therefore, we evaluated whether there were significant differences in antibody activity, thyroid function test (TFT), sonographic findings that may show the evolution of antithyroid antibody, TFT, and the sonographic score of the Sostre and Reyes classification system.

## Materials and Methods

Study Design

This descriptive cross-sectional study was conducted from January 2019 to May 2020 in patients who were referred to endocrinology clinics affiliated with the local Ethics Committee and Vice-Chancellor of Research at Shiraz University of Medical Sciences (SUMS) with a possibility of thyroid dysfunction and who were not under medical treatment.

Based on the statistical test for comparing means between two independent samples, a sample size of 142 subjects was calculated for each sample separately. The calculation considered a two-sided test with a type I error rate (α) of 0.05 and a desired power of 0.80. The population values used for the calculation were based on the Willms et al. [10] Study with a mean of 224.77 for the Homogeneous texture of thyroid, a mean of 374.5 for heterogeneous texture, and a common standard deviation of 450 (pooled approximately).

A total of 149 subjects (118 women, 31 men 18-60 years of age with a mean age of 38.60 ± 8.03 years) who met the criteria for the diagnosis of Hashimoto's thyroiditis were selected by simple consecutive sampling. Patients with a positive history of thyroid dysfunction or malignancy and those who consumed drugs that affected thyroid function (eg, thyroid hormones, thioamides, radioactive iodine, or oral contraceptive pills) and pregnant ones were excluded. This study was approved by the local Ethics Committee of the Shiraz University of Medical Sciences.

Setting

After explaining the study objectives, the participants were asked to sign a written informed consent. Then a trained nurse filled out a questionnaire containing questions about demographics, educational and marital status, individual habits, history of thyroid disease, and current medications. The weight was measured using a mechanical balance scale (Seka Vogel and Halke, Hamburg, Germany) with a precision of 0.5 kg. Height was measured to a precision of 0.5 cm in an upright position using a portable stadiometer (SECA stadiometer). Waist circumference was measured midway between the lower rib margin and the superior anterior iliac spine through a nonstretchable tape. Based on WHO classification criteria [9], clinical examination of the thyroid for the presence of goiter, thyroid consistency, presence or absence of the nodule, and their size was performed on the participants by an endocrinologist.

Laboratory Tests

Blood samples (10 ml) were collected in standard tubes for the measurement of free T3 (FT3) and FT4 (RIA, Immunotech, Czech, Ref. IM1579/IM3320), TSH (IRMA, Immunotech, Czech, Ref. IM3712/IM3713), anti-TPO and anti-Tg antibody titer (Competitive RIA, Immunotech, Czech, Ref. IM3712/IM3713) and sent to the Endocrine Research Center of the Nemazi Hospital affiliated with Shiraz University of Medical Sciences. All tests were performed with the same commercial kits. FT3, FT4, and TSH levels of 2.5 - 5.8 pmol/L, 11.5 - 23 pmol/L, and 0.17 - 4.05 mIU/mL were taken as normal values, respectively. The anti-TPO and anti-Tg antibody titer >60 IU/mL were considered positive. Subclinical hypothyroidism has been described as TSH>4.05 mIU / ml with normal FT4. Serum TSH level>4.05 mIU / ml and FT4<11.5 pmol/L is described as overt hypothyroidism.

Ultrasonography

According to standard ultrasound recommendations, the patients were placed supine with the neck in a slightly hyperextended position. Then scanning was performed in both transverse and longitudinal planes using a sonographic machine (HONDA ELECTRONIC HS-2200, Japan) with a 7.5 MHz transducer. Ultrasound examinations were performed by a thyroid ultrasound-trained sonographer for all patients. Various assessments were recorded [11]. The thyroid volume was calculated using the three-axis method. The thyroid lobes were considered elliptical, and the volume of each lobe was calculated using Brunn's formula (volume (ml)=length × width × thickness × 0/479) and the total thyroid volume from the total volume of the two lobes without the isthmus. The average normal volume of the thyroid gland in nongoiter subjects in men and women was reported to be 12-18 ml and 10-15 ml, respectively. Patients with thyroid volumes greater than these values were classified as having thyromegaly. Echogenicity was compared to adjacent strap muscles and classified as isoechoic and hypoechoic when its echogenicity was equal to or less than strap muscles. On the Reyes grayscale classification, thyroid nodules were divided into two macronodular and micronodular variants. Nodules were called micronodules when their size was ≤6 mm [12]. Based on analyses of grayscale sonographic characteristics, thyroid patterns were categorized according to the following scale (grading system created by Sostre and Reyes) [8]: (G0): Thyroid gland of normal size and echogenicity, (G1): Large diffuse gland with normal echo (similar to normal tissue ecology), (G2): Multiple hypoechoic foci or patches scattered throughout an otherwise normoechoic gland which is more indicative of focal rather than diffuse involvement (G3): Enlarged gland with diffuse but mild hypoechogenicity and (G4): Enlarged gland with diffuse and marked hypoechogenicity.

Data Analysis

Data were analyzed using a t-test, Chi-square, and one-way ANOVA. A nonparametric test (for data that did not have a normal distribution) of Kruskal Wallis was also performed using SPSS software (version 21, Chicago, IL). The correlation was evaluated by the Pearson correlation test. P-value<0.05 were considered significant.

Ethics Approval

The local Ethics Committee and Vice-Chancellor of Research at Shiraz University of Medical Sciences (SUMS) approved this study with the reference number IR.SUMS.REC.1395.S161. All patients and their parents signed a written informed consent form for participation in the study and any possible publication of their data, after explaining the aim, method and goal of the study to the participants.

## Results

**Figure-1:**
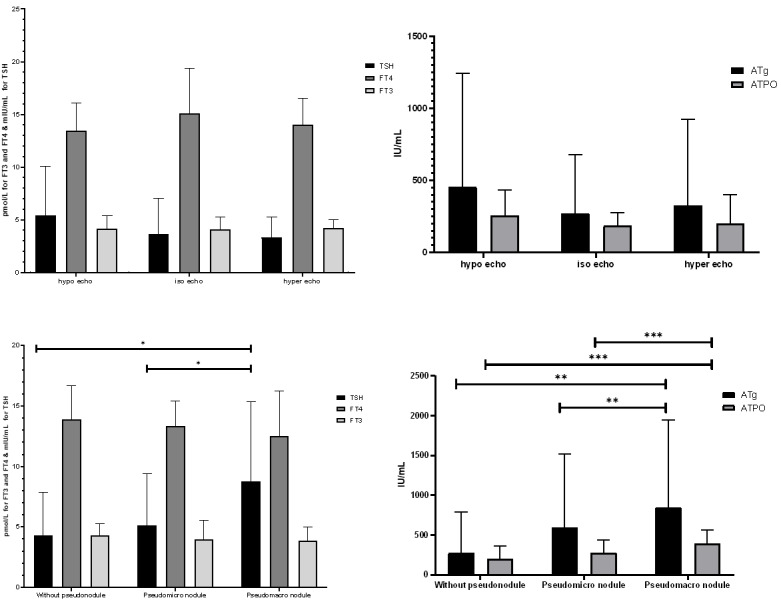
Figure 1. Comparison of the thyroid hormone and autoimmune antibodies based on the ultrasonographic findings. All tests are one way ANOVA with Tukey post hoc tests. * P<0.001; ** P<0.01; P<0.05.

**Table:** Table1. Sociodemographic and Clinical Characteristics of the Study Participants according to Thyroid Functional Status

Parameters		All n= 149	Overt Hypothyroidism n= 24	Subclinical Hypothyroidism n= 39	Euthyroid subjects n= 86	P-value*
Age, years, mean ± SD		38.6±8.03	38.57±8.46	37.18±8.35	41±5.16	0.181
Sex, male, n (%)		31(20.81%)	2(1.34%)	22(14.77%)	7(4.7%)	0.161
Marital status, married, n (%)		142(95.3%)	24(16.11%)	82(55.03%)	36(24.16%)	0.374
	Illiterate	22(14.77%)	4(2.68%)	13(8.72%)	5(3.36%)	
Educational status, n (%)	Elementary	120(80.54%)	20(13.42%)	69(46.31%)	31(20.81%)	0.598
	Higher Education	7(4.7%)	0(0%)	4(2.68%)	3(2.01%)	
Cigarette smoking history, n (%)		6(4.03%)	0(0%)	5(3.36%)	1(0.67%)	0.380
Water vaporizing smoke history, n (%)		30(20.13%)	5(3.36%)	13(8.72%)	12(8.05%)	0.129
Height, cm, mean ± SD		157.44±8.13	159.56±8.55	160.86±8.73	158.09±8.1	0.187
Weight, kg, mean ± SD		71.6±10.19	70.71±11.85	71.2±12.09	69.13±12.41	0.102

Continuous variables are compared by one way ANOVA with Tukey post hoc tests; Categorical data are compared by chi-square test.

**Table:** Table2. Ultrasound Features of the Study Participants according to Thyroid Functional Status

Parameters		All n= 149	Overt Hypothyroidism n= 24	Subclinical Hypothyroidism n= 39	Euthyroid subjects n= 86	P value
	Without pseudonodule	95(63.76%)	11(45.83%)	22(56.41%)	62(72.09%)	
Pseudonodule, n (%)	Pseudomicro nodule	38(25.5%)	6(25%)	12(30.77%)	20(23.26%)	0.008
	Pseudomacro nodule	16(10.74%)	7(29.17%)	5(12.82%)	4(4.65%)	
	Hypoechoic	119(79.87%)	22(14.77%)	34(22.82%)	63(42.28%)	
Echogenicity of parenchyma, n (%)	Hyperechoic	11(7.38%)	2(1.34%)	0(0%)	9(6.04%)	0.111
	Isoechoic	15(10.07%)	0(0%)	5(3.36%)	10(6.71%)	
	Solid	12(8.05%)	2(8.33%)	4(10.26%)	6(6.98%)	
Nodule Composition, n (%)	Cystic	2(1.34%)	1(4.17%)	0(0%)	1(1.16%)	0.785
	Complex	4(2.68%)	0(0%)	1(2.56%)	3(3.49%)	
Nodule Shape, n (%)	Globular	20(13.42%)	4(16.67%)	6(15.38%)	10(11.63%)	0.864
	Irregular	1(0.67%)	0(0%)	0(0%)	1(1.16%)	
Reactive lymph node, n (%)		2(1.34%)	5(20.83%)	1(2.56%)	2(2.33%)	0.702
	G0	28(71.81%)	11(54.17%)	8(66.67%)	9(79.07%)	
	G1	15(15.44%)	4(20.83%)	7(17.95%)	4(12.79%)	
Gray scale calcification, n (%)	G2	89(7.38%)	28(20.83%)	29(10.26%)	32(2.33%)	0.116
	G3	9(4.7%)	3(4.17%)	2(5.13%)	4(4.65%)	
	G4	8(0.67%)	3(0%)	3(0%)	2(1.16%)	
	thickness	15.72±3.72	15.04±3.1	14.8±2.94	15.14±3.03	0.132
	width	17.4±3.26	17.21±2.97	17.23±2.75	17.06±3.3	0.961
Right lobe, mm, mean ± SD	length	45.49±2.85	46.11±3.13	46.67±2.89	45.28±3.59	0.055
	Volume, ml	5.68±2.2	6.25±4.01	7.27±5.88	5.53±2.2	0.478
	thickness	15±3.24	14.3±3.02	14.03±3.02	14.46±2.88	0.544
	width	17.08±3.64	16.9±2.98	16.96±2.67	16.67±3.26	0.354
Left lobe, mm, mean ± SD	length	44.08±3.72	44.87±3.57	45.07±3.6	44.9±3.43	0.844
	Volume, ml	5.48±2.52	5.13±2.52	5.25±3.21	4.67±1.6	0.486
Isthmus thickness, mm, mean ± SD		1.86±0.68	1.91±0.9	1.77±0.74	2.26±1.2	0.018

Continuous variables are compared by one way ANOVA with Tukey post hoc tests; Categorical data are compared by chi-square test.

Baseline Sociodemographic and Clinical Characteristics of the Enrolled Patients

The analysis of the results showed that of 149 patients, 118 (79%) were women and 31 were men (21%); their mean age was 38.60±8.03 (range, 18-60) years (Table-1).

The results of the comparison of baseline sociodemographic and clinical characteristics between the normal thyroid function group and different thyroid dysfunction groups are shown in Table-1. Mean age, sex, marital status, educational status, smoking history, height, and weight did not differ between these subgroups (Table-1).

Ultrasound Features Concerning Thyroid Functional Status

The ultrasound characteristics of the patients were classified according to echogenicity and nodularity of the thyroid parenchyma. The sizes of the right and left thyroid lobes and isthmus in terms of length, width, and anterior-posterior diameter were summarized and compared by thyroid function status. The mean thyroid volume in the men in the study population was 13.58 ± 4.19 and in the women 10.44 ± 3.32 (P<0.05). Echogenicity was recorded as isoechoic in 15(10.07%) and hypoechoic in 119 subjects (79.87%). Nodular structures were detected in 54 (36.2%) patients, of whom 38 (25.5%) micronodular and 16 (10.7%) macronodular were detected. The length, width, thickness, and volume of the left and right thyroid lobes and total thyroid volume did not differ between the study groups (P>0.05). There was a significant difference in isthmus thickness between the study groups, where euthyroid ‎subjects had significantly thicker isthmus than overt and subclinical hypothyroid patients (P=0.018). Furthermore, the mean thyroid volume in the 149 subjects was 11.10 ± 3.73 ml. There were no statistically significant differences in the distribution of the nodule structures in the study groups (P=0>05). According to the Sostre and Reyes grayscale classification, the thyroid glands were scored as grade 0 in 28 patients, grade 1 in 15 patients, Grade 2 in 89 patients, Grade 3 in 9 patients, and grade 4 in 8 patients. There were no statistically significant differences in the distribution of nodular structures in the study groups (P= 0.116), as shown in Table-2.

Ultrasound Features about Thyroid Functional Parameters and Thyroid Autoimmune Antibodies

Figure-1 shows the changes in thyroid function tests and the titer of thyroid autoimmune antibodies according to the ultrasonographic findings. At baseline, mean serum levels of thyroid hormones, anti-TPO, and anti-Tg antibodies were compared more in the study groups. The serum TSH, anti-TPO, and anti-Tg antibody titers were significantly higher in patients with hypoechogenicity than in those with normal echogenicity (P=0.018, P=0. 035 and P=0.014, respectively). Mean serum TSH was significantly higher in patients with macronodules than in those with micronodules and subjects without nodules (P=0.003). The serum anti-Tg and anti-TPO antibody titers were significantly higher in macronodule patients than in those with micronodules and subjects without nodules (P<0.05).

Correlation of Thyroid Function Tests and Thyroid Measurements

**Figure-2:**
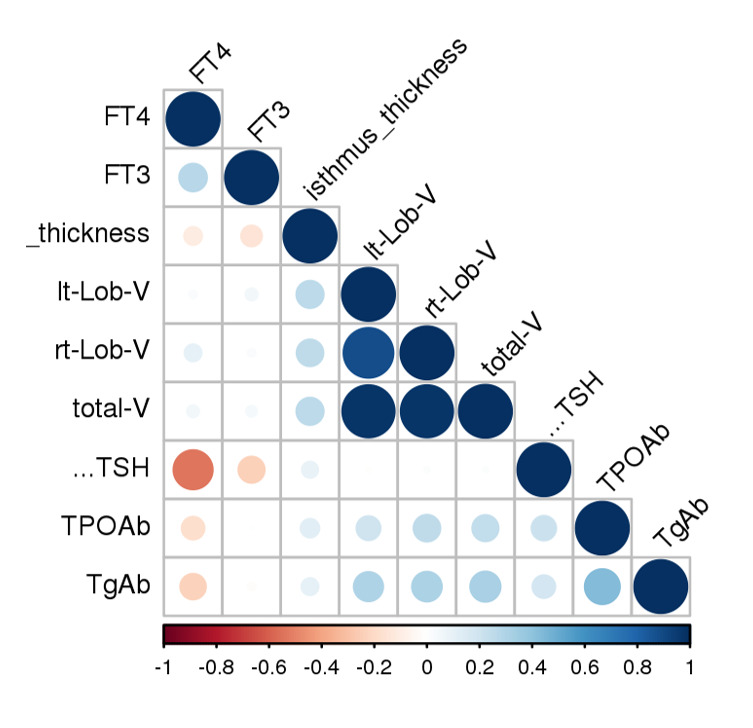
Figure 2. Correlogram of the relationship between serum thyroid factors and volume thyroid in the study subjects; the red circles show negative significant correlations and blue ones show positive correlations (the strength of the color shows the amount of the Pearson correlation (lt: Left, rt: Right, V: volume).

When we correlated the baseline sonographic findings with the thyroid function test, anti-TPO, and anti-Tg antibody titers, there were significant correlations, as shown in Figure-2. The thickness of the isthmus had a significant negative correlation with FT4 (P=0.046; r=0.11). FT3 also had a significant negative correlation with the isthmus thickness (P=0.017; r=0.15). The anti-TPO antibody titer had a significant positive correlation with the total volume (P=0.011; r=0.24). The anti-TPO antibody titer had a significant positive correlation with the right lobe volume (P=0.018; r=0.2). The anti-Tg antibody titer had a significant positive correlation with isthmus thickness (P=0.006; r=0.1). The anti-Tg titer had a significant positive correlation with left lobe volume (P=0.012; r=0.3). Additionally, it had a significant positive correlation with right lobe volume (P=0.019; r=0.31) and a significant correlation with total volume (P=0.042; r=0.32).

Grading of the Thyroid Ultrasonographic and Thyroid Function

The sonographic parameters examined in this study were reassigned to the Sostre and Reyes classification system. Most of the patients were in grade 2 and then in grade 0. There was a significant difference in serum anti-TPO titer among different thyroid grade scores. A posthoc test revealed a higher anti-TPO antibody titer in G4 than all other grades (P<0.05).

Furthermore, we compared various groups to detect significant differences in TSH, FT4, and FT3 concentration. According to Table-3, there were no significant differences between the parameters mentioned.

## Discussion

**Table:** Table3. The Result of the Serum Thyroid Factors in different Grades of Thyroid Ultrasonographic Indexes (G0- G4).

Variables	G0	G1	G2	G3	G4	P-value
Serum free T4 (pmol/L)	13.42±2.51	13.98±4.07	13.87±2.52	13.42±2.59	14.22±3.70	0.979*
Serum free T3 (pmol/L)	4.25±0.88	4.70±2.06	4.11±0.86	3.50±1.52	3.28±1.44	0.14*
Serum TSH (mIU/mL)	4.76±4.08	4.66±4.41	5.24±4.72	5.92±2.98	5.18±0.88	0.223*
TPO-Ab titer (IU/mL)	206.59±160.3	287.14±145.75	285.82±197.41	280.54±238.48	335.7±165.11	0.032**
Tg-Ab titer (IU/mL)	677.04±365.63	375.94±458.87	755.77±405.09	981.16±722.38	1321.7±769.01	0.301**

TSH thyroid stimulating hormone, Tg-Ab anti-thyroglobulin, TPO-Ab anti-thyroid peroxidase antibody. ^*^ Variables are compared by one way ANOVA with Tukey post hoc tests. ^**^ Variables are compared by Kruskal Wallis.

With the advent of more precise diagnostic tests, the diagnosis of Hashimoto's thyroiditis cannot be trusted merely on a single diagnostic procedure [13]. The histopathological diagnosis of Hashimoto’s thyroiditis is made following microscopic identification of chronic lymphocytic thyroiditis. However, since most patients do not undergo thyroidectomy, in the clinical setting, the diagnosis is made through the detection of elevated serum anti-TPO Ab and anti-Tg Ab antibodies [14]. When a patient presents with an undiagnosed thyroid pathological condition, an ideal diagnostic test should ideally be able to suggest the presence of thyroid autoimmunity, distinguish it from other thyroid illnesses, measure organ size and shape, detect changes in size during follow-up, and provide information about disease activity. The US of the thyroid has considerable advantages, including its availability at the bedside, ease of use, and reproducibility; It has been proven to be very effective in the diagnostic approach to thyroid disorders [15].

Some characteristics of the US are associated with developing hypothyroidism. Most of the available data on diagnostic studies in the setting of Hashimoto’s thyroiditis comes from those who already receive levothyroxine replacement therapy [16]. The purpose of this study was to evaluate the role of sonography in assessing functional disorders of the thyroid in a group of patients with Hashimoto's thyroiditis. We suggest that sonographic patterns demonstrate the type and extent of structural changes of the thyroid in different titers of thyroid antibodies and thyroid function. Thyroid autoantibodies had positive significant correlations with different parameters of thyroid volume. The results of our study showed that the increase in thyroid volume was consistent with the increase in anti-TPO antibody titers. In general, the role of anti-TPO antibodies in the tissue destruction process associated with hypothyroidism due to Hashimoto's thyroiditis has been proven, and its cytotoxic effects on thyroid cells accelerate thyroid dysfunction due to complement fixation strength [5].

Furthermore, it has a higher pathological value than the anti-Tg antibody titer and is more specific and more valuable for the diagnosis of autoimmune thyroid disorders [17]. On the other hand, thyroid enlargement is due to inflammation and cell infiltration of thyroid tissue during illness. Therefore, this study showed that increased thyroid volume was associated with a higher anti-TPO antibody titer. Hypoechogenecitis is a known phenomenon in Hashimoto's thyroiditis. As mentioned above, hypoechogenicity of the thyroid can be due to infiltration of the lymphocytic tissue. The decreased echogenicity of the thyroid may be due to a reduction in the content of the parenchymal thyroid colloid, an increase in thyroid blood flow, or infiltration of the lymphocytic tissue [18].

Our study is consistent with other studies, showing that the chances of developing hypoechogenicity increase significantly with increasing TSH, anti-Tg antibody, and anti-TPO antibody titers [16]. Of the few studies that evaluated the relationship between anti-Tg antibody titer and hypoechogenicity, none has found any such association [19][20]. It is still arguable whether all patients with autoimmune thyroid diseases are at increased risk for nodules and thyroid cancer or whether certain thyroid characteristics increase this risk [21].

In our study, mean serum TSH, anti-Tg antibody and anti-TPO antibody titers were significantly higher in patients with macronodules than those with micronodules and subjects without nodules. The formation of pseudo-nodules in thyroid tissue can be commonly found in Hashimoto's thyroiditis. Due to inflammation and infiltration of immune cells in thyroid tissue, the chance of forming pseudonodules in thyroid tissue increases. Infiltration of immune cells follows an increase in the activity of the autoimmune system. Anti-TPO and anti-Tg antibodies play a role as autoimmune-specific markers, so obviously the chance of thyroid nodularity increases with the presence of an increase in the titers of autoantibodies. Hypoechoic pseudonodular and multifocal lesions are likely to represent areas of high inflammatory activity and lymphocytic infiltration.

Reduced echogenicity results in reduced colloid content, increased thyroid blood flow, or increased lymphocytic infiltration. Inflammatory status promotes the development of thyroid nodules, perhaps due to its indirect effect of hindering the synthesis of thyroid hormone, which results in the elevation of TSH [19]. Many thyroid growth stimulating factors, such as TSH, insulin-like growth factor-1, and fibroblast growth factor, might be involved in the development of adenomatous lesions in patients with Hashimoto’s thyroiditis [22][23].

One hundred twenty-five (85.9%) patients in our study had subclinical hypothyroidism or a euthyroid state. Subclinical hypothyroid patients with underlying thyroid disease have an increased risk of developing overt hypothyroidism, which is associated with adverse effects on lipid profile and cardiovascular function [24].

However, predicting disease progression and assessing the risk of evolution to a more severe form of thyroid dysfunction is challenging. In our study, based on the classification system published by Sostre and Reyes, the US pattern of the thyroid was found in most of our patients in the G2 class. Therefore, most patients suffering from Hashimoto's thyroiditis had a sonographic thyroid pattern consisting of multiple hypoechoic foci or patches scattered throughout an otherwise normoechoic gland, which is more indicative of focal rather than diffuse involvement. The G2 pattern is more likely to indicate mild to moderate thyroid involvement, which is more common in patients with more subclinical symptoms. Furthermore, the results of the present study showed that the highest Anti-TPO antibody titers were in G4. The anti-TPO antibody could cause a defective thyroid organization and shift the surface area of the thyroid structure and the thyroid ultrasound pattern of the thyroid toward higher grades.

Limitations of Study

There were some limitations in this study; it used a cross-sectional design and included a relatively small number of participants who underwent US examinations in a single institution. Additionally, we did not conduct a follow-up ultrasound of consecutive patients. A study with a larger sample size and follow-up should be conducted to validate our results. Our study ultrasonographic assessments were conducted by a single physician which might have biased study results and there should be more observers in future studies for evaluation of inter-observer agreement. As another limitation, the extent of dose and time passing the initiation of the levothyroxine treatment causes fluctuations in the TSH, FT3, and FT4 levels that make it impossible to consider all these confounding factors in a cross-sectional study, so we excluded patients who were previously treated for Hashimoto.

## Conclusions

Our findings suggest that elevated levels of anti-TPO antibodies may lead to notable alterations in the US thyroid markers, potentially due to the disruptive impact of this autoantibody on thyroid organization. Consequently, integrating US evaluation with the assessment of anti-TPO and anti-Tg antibody titers could prove beneficial in identifying and investigating the severity and extent of Hashimoto's thyroiditis. This combined approach may assist in identifying patients at greater risk of developing hypothyroidism, facilitating timely and regular follow-up care.

## Acknowledgments

The authors would like to thank Shiraz University of Medical Sciences, Shiraz, Iran, and also the Center for Development of Clinical Research of Nemazee Hospital and Dr. Nasrin Shokrpour for editorial assistance. A preprint has previously been published (https://www.researchsquare.com/article/rs-835595/v1). This study was supported by the Shiraz University of Medical Sciences [IR.SUMS.REC.1395.S161].

## Conflicts of Interest

The authors declare that they have no competing interests. Authors disclose all relationships or interests that could have direct or potential influence or impart bias in the work.
